# REFRAMING AGING FOR HIGHER EDUCATION IN HEALTH CARE PROFESSIONS

**DOI:** 10.1093/geroni/igad104.3772

**Published:** 2023-12-21

**Authors:** Caitlyn Coyle, Cindy Bui, Setarreh Massihzadegan, Suha Ballout, Jan Mutchler

**Affiliations:** University of Massachusetts Boston, Boston, Massachusetts, United States; University of Massachusetts Boston, Boston, Massachusetts, United States; University of Massachusetts Boston, Boston, Massachusetts, United States; University of Massachusetts Boston, Boston, Massachusetts, United States; University of Massachusetts Boston, Boston, Massachusetts, United States

## Abstract

As students in health care professions will serve older patients, it is critical to proactively reframe aging in their education, confront implicit biases, and encourage age-inclusivity before they enter the workforce. We developed a training to address the limited focus on aging and later life among undergraduate curricula in health care professional disciplines (e.g., nursing, health sciences). The 1-hour, online training was piloted among current undergraduate nursing students (N=69). The content uses data and real-person interviews to introduce demographic trends in aging, define and deconstruct ageism concepts, illustrate the impact of ageism on individual health and health care, and highlight the diverse experiences of aging. The training also includes communication tools and interactive activities, such as reflection questions and case studies, to engage students in their own aging experience and to garner empathy for others’ aging experiences. Pre- and post-training evaluations revealed that the training impacted students in expressing positive behavioral change towards age-inclusivity and awareness at internal, interpersonal and institutional levels. Evaluation results also showed statically significant evidence that the training increased students’ knowledge about facts versus stereotypes about aging, confidence in understanding ageism and its relevance to health care, and consideration of working with older adults in their career. This pilot research project encourages other higher education institutions to incorporate training and education in their curricula that distinctively focuses on later life and reframing aging in a long-term effort towards improving health care for older people and the experiences of health care professionals who serve them.

